# Exacerbating Guillain–Barré Syndrome Eight Days after Vector-Based COVID-19 Vaccination

**DOI:** 10.1155/2021/3619131

**Published:** 2021-05-08

**Authors:** Josef Finsterer

**Affiliations:** Klinik Landstrasse, Messerli Institute, Postfach 20, Vienna, Austria

## Abstract

Since the introduction of mRNA technology-based and vector-based COVID-19 vaccines, adverse reactions to these agents have been occasionally reported. Exacerbation of Guillain–Barré syndrome (GBS) shortly after COVID-19 vaccination has not been communicated. The patient is a 32-year-old male who developed progressive sensory disturbances and muscle weakness 8 days after the first dosage of a vector-based vaccine. Cerebrospinal fluid investigations revealed a dissociation cyto-albuminque, and nerve conduction studies revealed demyelination. Intravenous immunoglobulin (IVIG) exhibited only a marginal effect for both sensory and motor deficits. The patient's history was moreover positive for previous GBS with marked motor deficits 14 years earlier, which responded favourably to IVIG leading to almost complete recovery within 9 months of rehabilitation. Although apparently extremely rare, neurologists should remain vigilant for a potential recurrence of GBS after vaccination with a vector-based COVID-19 vaccine.

## 1. Introduction

Since the introduction of COVID-19 vaccines, adverse reactions have been occasionally reported [1]. Whether the frequency of adverse reactions varies between mRNA technology-based and vector-based COVID-19 vaccines has not been investigated. We herein report a patient who developed Guillain–Barré syndrome (GBS) 8 days after the first vaccination with a vector-based COVID-19 vaccine. Verbal consent was obtained from the patient to publish the report.

## 2. Case Presentation

The patient is a 32-year-old male who developed paresthesia on both foot soles two days prior to admission (hospital day (hd)-2), followed by paresthesias of both palms and dysphagia one day later (hd-1). Additionally, he reported bilateral frontal and nuchal headache on hd-1 scoring 6 on the visual analogue scale (VAS). Headache improved to VAS 3 on hd-1 after ibuprofen. The individual's history was positive for a first vaccination against COVID-19 with a vector-based COVID-19 vaccine 8 days prior to onset of sensory disturbances, respectively, 10 days prior to admission. In the night after vaccination, the patient experienced chills, headache, and body aches and measured a body temperature of 37.8°C, which completely resolved on the next day without treatment. The history was moreover positive for previous GBS 14 years earlier after a gastrointestinal infection manifesting with quadruparesis, dysphagia, and facial diplegia but without involvement of the respiratory muscles. After application of intravenous immunoglobulin (IVIG) and rehabilitation during 9 months, the deficits almost completely resolved. Moreover, the patient conceded occasionally taking amphetamines, which he received on the black market as a powder. The family history was positive for bipolar disorder and a brain tumour of unknown dignity in his mother.

Clinical neurologic exam on admission (hd1) revealed a discrete, right-sided signe des cils (residual), discrete right-sided peripheral facial palsy affecting the orbicularis oris muscle (residual), bilateral muscle weakness (M5−) with elbow extension, reduced tendon reflexes on the upper limbs, absent patella tendon reflexes, an absent right-sided Achilles tendon reflex, and a reduced Achilles tendon reflex on the left side. The patient denied any sensory disturbances on clinical testing. Blood tests were normal. Panels for vasculitis, rheumatic, and autoimmune diseases were noninformative. Urine toxicology was positive for cannabinoids. Otolaryngological investigation was noninformative. MRI of the brain and cervical spine revealed a few nonspecific T2-hyperintensities in the white matter bilaterally. Investigation of the cerebrospinal fluid (CSF) on hd1 revealed a CSF protein of 58.4 mg/dl (*n*, 20–40 mg/dl). On hd2, the patient reported sensory disturbances of the tongue tip in addition to previously recognised paresthesias and muscle weakness for elbow extension. IVIG (0.4 g/kgBW) was started on hd2. Nerve conduction studies on hd3 revealed slowed nerve conduction velocity, prolonged distal latencies, and absent F-wave responses. On hd3, right-sided facial palsy slightly progressed. On hd34, he presented with facial diplegia, dysarthria, weakness for elbow extension (M5−)/flexion (M4+), and weakness for hip flexion (M5−) and foot extension (M5−). Plasmapheresis improved the condition but after nosocomial infection, muscle weakness and dysarthria deteriorated and dysphagia newly developed.

## 3. Discussion

The patient is interesting for developing GBS 8 d after vaccination with a vector-based COVID-19 vaccine. Whether the relation between vaccination and GBS was causal or coincidental remains speculative. Arguments for a causal relation between vaccination and GBS are that the patient had developed a flu-like illness a few hours after vaccination, that no other infectious focus could be identified, and that there was a temporal relation between vaccination and the onset of GBS. Arguments against a causal relation are that a causal relation has not been reported and that SARS-CoV-2 vaccinations are usually well tolerated. However, there are a few reports about class 1–4 adverse advents after COVID-19 vaccination [1, 2]. The most common adverse events in a study of 21,977 patients who received a vector-based vaccine in Russia were flu-like illness, injection site reactions, headache, and asthenia [1]. Adverse reactions to mRNA-based vaccines reported include allergetic reactions [2], supraclavicular lymphadenopathy (postvaccine adenopathy) [3], radiation recall phenomenon [4], or Bell's palsy [5]. According to the current literature, the risk for GBS after COVID-19 is higher than the risk after vaccination, similar to influenza [6]. Additionally, there is consensus that the benefits of vaccination outweigh the risks of COVID-19 infection and associated morbidity and mortality [7]. Whether the findings on NCSs, carried out on hd3, represent residual abnormalities from the first GBS or have newly developed together with the second GBS remains speculative. Unfortunately, the patient had not undergone follow-up NCS between the first and second GBS. Overall, there is a need to further improve the diagnosis and therapy of GBS regardless of the causative trigger [8].

## 4. Conclusion

This case shows that vaccination with a vector-based COVID-19 vaccine can be followed by exacerbation of GBS. To establish an eventual causal relation, further studies about the toxicity of COVID-19 vaccines are warranted.

## Data Availability

All data are available from the corresponding author upon request.
